# Machine learning models integrating 2D and 3D intratumoral heterogeneity features from multi-sequence MRI: accurate prediction of perineural invasion in pancreatic ductal adenocarcinoma

**DOI:** 10.3389/frai.2026.1975752

**Published:** 2026-10-20

**Authors:** Zhiqiang Ouyang, Zhengrui Song, Zhilin Li, Shulan Cui, Xin Liu, Wan Shen, Fangyu Zhu, Chengde Liao, Wanjun Hu, Shaonan He, Yi Lu, Qiuwen Ye

**Affiliations:** 1 Department of Radiology, Yan’an Hospital of Kunming City (Yanan Hospital Affiliated to Kunming Medical University), Kunming, China; 2 Key Laboratory of Tumor Immunological Prevention and Treatment of Yunnan Province, Kunming, China; 3 Hepatobiliary and Pancreatic Surgery, Yunnan Cancer Hospital (The Third Affiliated Hospital of Kunming Medical University), Kunming, China; 4 Department of Medical Imaging, Yunnan Cancer Hospital (The Third Affiliated Hospital of Kunming Medical University), Kunming, China; 5 Department of Pathology, Yunnan Cancer Hospital (The Third Affiliated Hospital of Kunming Medical University), Kunming, China; 6 Department of Nuclear Magnetic Resonance, The Second Hospital and Clinical Medical School, Lanzhou University, Lanzhou, China; 7 Department of Medical Imaging, The First People’s Hospital of Yunnan Province (The Affiliated Hospital of Kunming University of Science and Technology), Kunming, China; 8 Department of Medical Imaging, The First Affiliated Hospital of Kunming Medical University, Kunming, Yunnan, China

**Keywords:** intratumoral heterogeneity, machine learning, magnetic resonance imaging, pancreatic ductal adenocarcinoma, perineural invasion

## Abstract

**Introduction:**

Perineural invasion (PNI) occurs in the majority of pancreatic ductal adenocarcinoma (PDAC) cases and is strongly associated with local recurrence, neuropathic pain, and poor survival, yet no reliable method exists for its preoperative identification. Here, we developed and externally validated a multi-sequence MRI-based intratumoral heterogeneity (ITH) scoring framework integrated with machine learning for the non-invasive prediction of PNI in PDAC.

**Methods:**

Using preoperative T2-weighted, diffusion-weighted, and contrast-enhanced T1-weighted MRI from 205 surgically resected patients across four medical centers (100 training, 43 internal test, and 28 and 34 external validation patients), we extracted two-dimensional and three-dimensional ITH features via K-means clustering-based subregion partitioning and incorporated independent clinical predictors tumor location and clinical T-stage to construct combined predictive models. Eleven machine learning algorithms were systematically evaluated.

**Results:**

The combined clinical-ITH model achieved an area under the curve (AUC) of 0.907 in the internal test set and AUCs of 0.860 and 0.869 in the two external validation cohorts. Corresponding AUCs were 0.881, 0.860, and 0.850 for the ITH model and 0.758, 0.342, and 0.700 for the clinical logistic regression model.

**Discussion:**

SHAP analysis ranked three T2WI-derived features as the largest contributors to the combined model output, feature effects varied in direction and were not uniformly monotonic. These findings demonstrate that multi-sequence MRI-based ITH quantification provides an interpretable approach to preoperative PNI assessment with consistent discrimination across the two external surgical cohorts, offering a potential tool to guide surgical planning and adjuvant therapy selection in pancreatic cancer.

## 1 Introduction

Pancreatic ductal adenocarcinoma (PDAC) remains one of the most lethal solid malignancies, with a 5-year survival rate below 13% despite significant advances in surgical techniques and systemic therapies (Siegel et al., 2024; Bray et al., 2024). A pathological hallmark of PDAC is perineural invasion (PNI), defined as the infiltration of tumor cells into the epineurium, perineurium, or endoneurium of peripheral nerves. PNI is detected in 70.8–93% of resected PDAC specimens and is recognized as a critical driver of local recurrence, postoperative neuropathic pain, and impaired overall survival (Schorn et al., 2017; Gasparini et al., 2025). Preoperative identification of PNI status carries substantial clinical implications: patients with PNI-positive disease may benefit from extended retroperitoneal dissection or intensified neoadjuvant therapy (Springfeld et al., 2026), whereas PNI-negative patients could avoid the morbidity associated with overtreatment. However, PNI can only be definitively confirmed through postoperative histopathological examination (Li et al., 2026b), leaving surgeons without this critical information during preoperative decision-making.

Conventional imaging, including contrast-enhanced computed tomography (CT) and magnetic resonance imaging (MRI), provides essential information regarding tumor size and vascular involvement (Qu et al., 2022; Srinivas Rao et al., 2026), yet remains insensitive to the microscopic neural infiltration characterizing PNI. This diagnostic gap has motivated radiomics—the high-throughput extraction of quantitative imaging features—to capture tumor phenotypes that escape visual assessment. Following the seminal demonstration that CT-derived radiomic features carry independent prognostic information (Aerts et al., 2014; Bera et al., 2022), radiomics-based approaches have been developed for predicting lymph node metastasis and histological grade in pancreatic neuroendocrine tumors (Bian et al., 2023; Mori et al., 2023; Gu et al., 2023), as well as vascular involvement in PDAC (Rigiroli et al., 2021). For PNI prediction specifically, CT-based radiomics and deep learning on super-resolution MRI have achieved moderate to good accuracy (Lan et al., 2026; Sun et al., 2025; Li et al., 2026a), yet these approaches either rely on hand-crafted global descriptors or end-to-end representations that fail to explicitly capture the spatial architecture of intratumoral subregions, a feature hypothesized to be biologically relevant to neural tropism.

Intratumoral heterogeneity (ITH), defined as the spatial and functional diversity of cells within a single tumor mass, has emerged as a central concept linking imaging phenotypes to tumor behavior (Alizadeh et al., 2015). In PDAC, ITH is particularly pronounced owing to the complex interplay between malignant epithelial cells, cancer-associated fibroblasts, immune infiltrates, and neural elements within the desmoplastic stroma (Öhlund et al., 2017; Caligiuri and Tuveson, 2023). ITH arises from clonal evolution driven by genomic instability and selective pressures within the tumor microenvironment (Black and McGranahan, 2021; Bailey et al., 2016). Radiogenomic analyses have linked imaging heterogeneity to underlying genomic diversity and patient prognosis (Su et al., 2023; Udayakumar et al., 2021), while spatial partitioning of tumors on MRI via unsupervised clustering has enabled quantitative ITH indices that predict treatment response (Shi et al., 2023). Multi-sequence MRI, which probes tissue diffusivity through DWI and captures tumor morphology and microenvironment characteristics through T2WI and T1CE (Palombo et al., 2026; Hoffmann et al., 2024), may therefore offer a powerful yet unexplored approach to PNI prediction in PDAC (Zhao et al., 2025).

Here, we developed and validated a multi-sequence MRI-based ITH scoring framework integrated with machine learning for the preoperative prediction of PNI in PDAC. Using a validated pixel and voxel-level clustering approach, we quantify spatial heterogeneity across T2WI, DWI, and T1CE in both two and three dimensions, generating a multi-dimensional ITH feature set that captures both the magnitude and spatial architecture of intratumoral heterogeneity. We systematically evaluate 11 machine learning algorithms, compare single-sequence and multi-sequence models, assess the incremental value of clinical predictors through a combined modeling strategy, and rigorously validate the framework across four independent medical centers (Figure 1).

**Figure 1:**
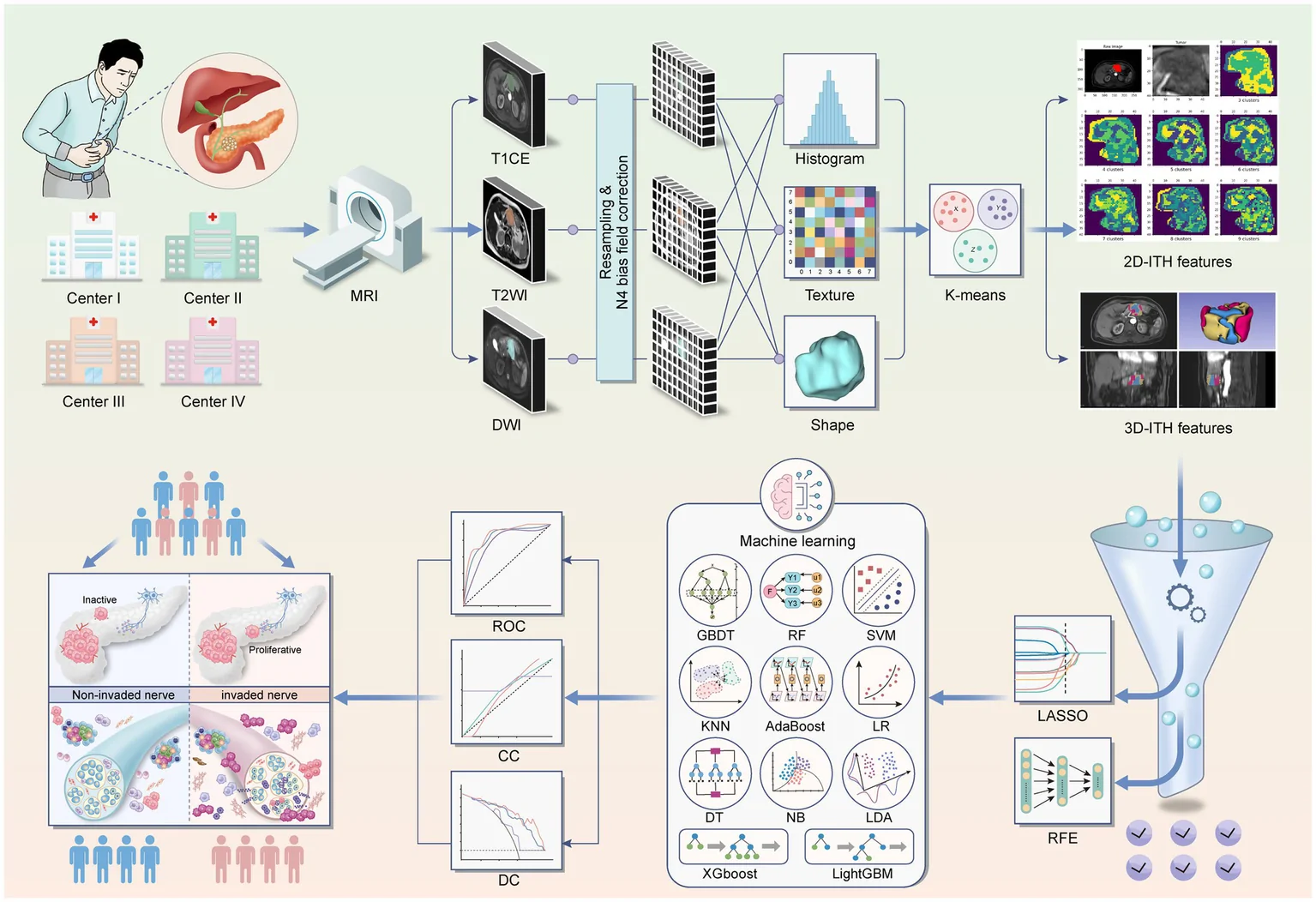
Overview of the study workflow. Pre-treatment multi-sequence MRI images of pancreatic cancer patients who visited four different hospitals were collected. After ROI segmentation and image processing, features related to 2D and 3D tumor heterogeneity scores were extracted via K-means unsupervised clustering analysis. Meanwhile, clinical features were incorporated. After feature selection, 11 machine learning algorithms were used to construct models capable of identifying perineural invasion in pancreatic cancer. Finally, through model evaluation, the model with the optimal diagnostic efficacy was selected.

## 2 Methods

This multi-center retrospective study received approval from the Research Ethics Committees of the Institutional Review Boards (approval numbers: KYLX2026-107 and 2026–148-01), and the requirement for informed consent was waived in accordance with institutional guidelines and the Declaration of Helsinki. This study was designed using the Transparent Reporting of a multivariable prediction model for Individual Prognosis or Diagnosis (TRIPOD) guidelines.

### 2.1 Patients

We retrospectively screened consecutive patients diagnosed and treated for pancreatic cancer across four independent medical centers. Patients admitted to Center I (the Third Affiliated Hospital of Kunming Medical University) between June 2019 and June 2024, and to Center II (the First Affiliated Hospital of Kunming Medical University) between June 2020 and June 2025, were pooled and randomly divided into training (70%) and internal test (30%) sets, as described in Section 2.6. Patients from Center III (the First People’s Hospital of Yunnan Province) between June 2019 and June 2024, and from Center IV (Yan’an Hospital of Kunming City) between June 2020 and June 2025, were enrolled as the first and second external validation cohorts, respectively. Inclusion criteria were as follows: (I) histopathological confirmation of pancreatic cancer via surgical resection; (II) explicit documentation of PNI status in the formal histopathology report; and (III) completion of abdominal MRI, including unenhanced scanning, DWI, and T1CE imaging, within one month before surgical resection. Exclusion criteria were as follows: (I) coexistence of other malignant tumors; (II) unavailable pathological information required to confirm the diagnosis or determine PNI status; (III) absence of any required MRI sequence; and (IV) poor image quality precluding diagnostic evaluation. The same exclusion categories are presented in Figure 2.

**Figure 2:**
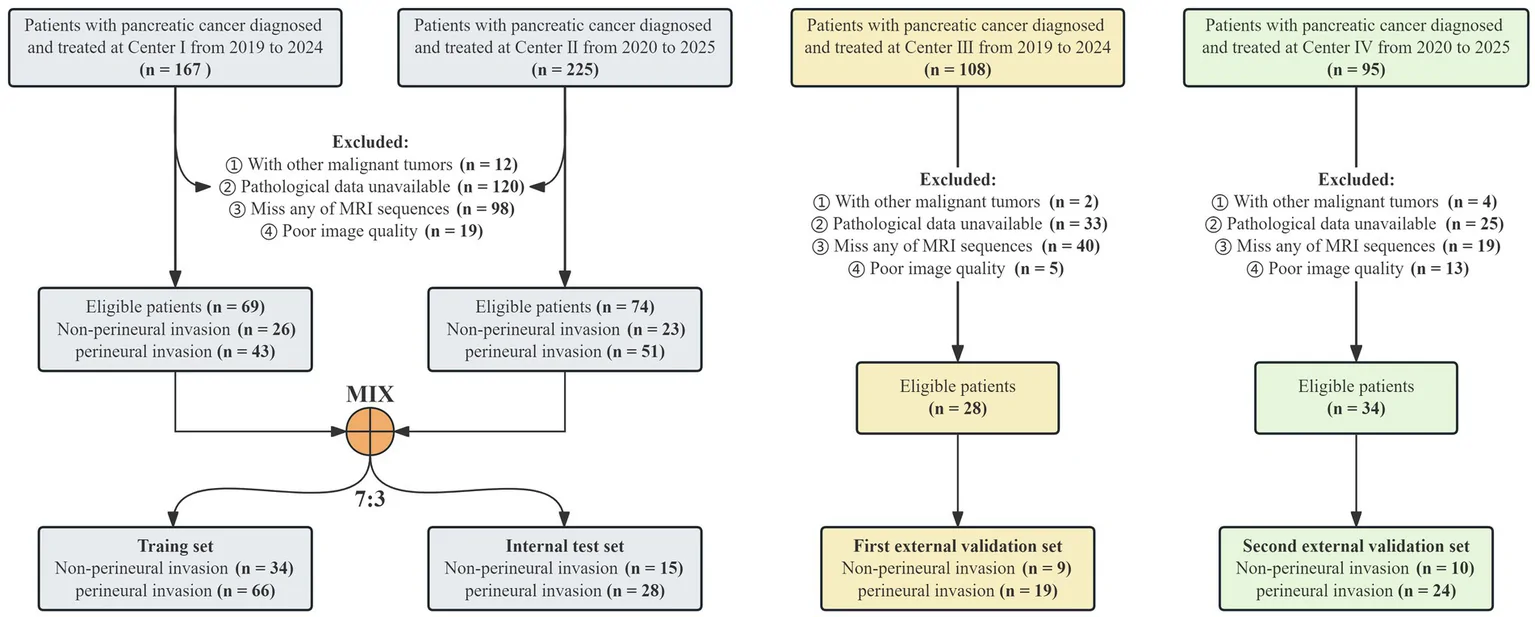
The flow diagram of patient inclusion process.

### 2.2 Pathological diagnosis of PNI

All surgically resected pancreatic specimens were fixed in 10% neutral buffered formalin for 24–48 h, processed using standard histological techniques, and embedded in paraffin. Serial 4-μm sections were cut and stained with hematoxylin–eosin (H&E) for routine examination. PNI was evaluated by two independent, board-certified pathologists blinded to clinical outcomes, using established criteria defined by the World Health Organization (WHO) and the College of American Pathologists (CAP) (Demir et al., 2015). PNI was diagnosed when tumor cells formed a circumferential cuff around more than one-third of the nerve circumference or invading any layer of the nerve sheath, including the epineurium, perineurium, or endoneurium. Discrepancies between pathologists were resolved through consensus review with a third senior pathologist.

### 2.3 MRI acquisition and preprocessing

The MRI sequences employed for feature extraction related to ITH scoring included abdominal axial T2WI, DWI, and T1CE. MRI scanning equipment and protocols varied across the four medical centers; detailed specifications for devices, sequences, and parameters are provided in Supplementary Table S1. To reduce differences in image geometry and intensity inhomogeneity, all images were preprocessed using Python software (version 3.7; https://www.python.org). Specifically, isotropic resampling was performed to standardize voxel dimensions to 1 × 1 × 1 mm^3^, followed by application of the N4 bias field correction algorithm to mitigate artifacts associated with magnetic field inhomogeneity.

### 2.4 Tumor segmentation

All images were reviewed by two board-certified radiologists (Reader 1 with 8 years of experience; Reader 2 with 10 years of experience), who were blinded to the pathological diagnosis of PNI. Volumes of interest (VOIs) were manually delineated on the solid component of the pancreatic tumor on each sequence, carefully excluding necrotic areas, cystic components, and major blood vessels. Inter-observer agreement was assessed using the intraclass correlation coefficient (ICC; two-way random, absolute agreement), and only features with ICC > 0.80 were retained for subsequent analysis.

### 2.5 ITH quantification

Both 2D and 3D intratumoral heterogeneity analyses were performed independently for each MRI sequence using a validated ITHscore framework (ITHscore Python package; Shi et al., 2023).

#### 2.5.1 2D ITH score

For each sequence, the axial slice containing the largest tumor cross-sectional area was semi-automatically selected as the representative region of interest (ROI). Each pixel within the ROI was assigned as the center of a local subregion, from which 104 radiomic features—comprising 10 shape-based feature, 19 first order statistics features and 75 texture features were extracted using PyRadiomics (version 3.1.0, https://github.com/Radiomics/pyradiomics). K-means clustering was applied to partition pixels into K discrete subregions based on feature similarity (robustness was further assessed across K = 3–9). For each cluster, the number of spatially contiguous connected components (nᵢ) and the area of the largest connected region (Sᵢ,max) were determined. The 2D ITH score was computed as: ITH = 1 − (1/S_total) × Σᵢ=₁ᴷ (nᵢ · S_ᵢ,max/n_i). Where S_total denotes the total tumor area. A higher score reflects greater spatial fragmentation of cluster labels and, consequently, more pronounced intratumoral heterogeneity.

#### 2.5.2 3D ITH metrics

The same radiomic feature extraction and K-means clustering pipeline was extended to three dimensions. Each voxel within the VOI served as the center of a 3 × 3 × 3 cubic neighborhood, yielding a voxel-level feature matrix on which clustering was performed. The 3D ITH score was calculated analogously to the 2D formulation, replacing area with volume (V_total and Vᵢ,max). In addition, 15 supplementary 3D heterogeneity indicators were derived to capture complementary aspects of volumetric and spatial complexity: original and volume-weighted ITH scores, maximum subregion proportion, subregion volume standard deviation and coefficient of variation, maximum/minimum volume ratio, Shannon entropy, Gini index, volume entropy, intensity heterogeneity index, intensity range (MIR), ecological imbalance index (EII), distribution shift index (DSI), volume-weighted fractal dimension, and fractal dimension variability index. Collectively, the 2D and 3D analyses yielded a multi-dimensional ITH feature set per patient across all three MRI modalities.

### 2.6 Feature selection and analysis

Patients from Centers I and II were pooled and further split into a training set (70%) and an internal test set (30%) using stratified random sampling based on the PNI label, ensuring balanced class distribution across splits. All features were standardized using Z-score normalization before analysis. A two-stage feature selection strategy, combining least absolute shrinkage and selection operator (LASSO) regression with recursive feature elimination (RFE) (Xiong et al., 2025), was applied to the training set to identify the most informative features while mitigating overfitting. In the first stage, LASSO logistic regression with 5-fold cross-validation (L1 penalty, SAGA solver, balanced class weights) was independently performed on each modality-specific feature subset. Features with non-zero LASSO coefficients were retained. In the second stage, a random forest-based RFE (100 estimators, maximum depth of 5, minimum of 5 samples per leaf, balanced class weights) was applied to the pooled LASSO-selected features to further reduce dimensionality. Univariate and multivariate logistic regression analyses were performed on baseline clinical characteristics, including preoperative imaging-based clinical T-stage (cT-stage), to identify independent predictors of perineural invasion. Variables achieving significance in the multivariate model were subsequently incorporated into the combined model.

### 2.7 Classifier development and hyperparameter optimization

Eleven binary classification algorithms were independently developed: logistic regression (LR), random forest (RF), support vector machine (SVM), extreme gradient boosting (XGBoost), light gradient boosting machine (LightGBM), k-nearest neighbors (KNN), decision tree (DT), gradient boosting decision tree (GBDT), adaptive boosting (AdaBoost), naive Bayes (NB), and linear discriminant analysis (LDA). For each algorithm, hyperparameter optimization was conducted via 5-fold stratified cross-validation with grid search on the training cohort, using the AUC as the optimization metric. To mitigate class imbalance, algorithm-specific strategies were employed: balanced class weighting for LR, RF, SVM, and DT; the scale_pos_weight parameter (set to the negative-to-positive sample ratio) for XGBoost; and the is_unbalance flag for LightGBM. Overfitting was controlled through regularization measures appropriate to each algorithm, including L1 and L2 penalties, tree-depth constraints, and subsampling ratios. To systematically identify the model with the highest diagnostic performance, we constructed prediction models using three individual MRI sequences and three combined multi-sequence feature sets, enabling a comprehensive comparison from single-sequence to multi-sequence approaches.

### 2.8 Statistical analysis and model evaluation

Age and serum tumor marker concentrations (CA19-9, CEA, and CA125) were summarized as mean ± standard deviation (SD). Age was compared between the PNI and non-PNI groups using an independent-samples Student’s *t*-test. Differences in mean CA19-9, CEA, and CA125 concentrations were assessed using two-sided Welch’s independent-samples *t*-tests, which do not assume equal variances. Maximum tumor diameter was summarized as the median (25th–75th percentiles) and compared using a Mann–Whitney U test. Categorical variables were summarized as counts and percentages and compared using chi-square or Fisher’s exact tests, as appropriate. The minimum sample size was calculated using binormal ROC curve analysis in PASS software (version 2026; https://www.ncss.com/). Model performance was evaluated using AUC, accuracy, sensitivity, specificity, precision, and F1 score, visualized in radar plots. The AUC with 95% confidence interval (CI) was estimated using bootstrap resampling (1,000 iterations). ROC curves were generated for each classifier across all four datasets, with smoothing applied via linear interpolation to improve visual clarity. Calibration performance was assessed using calibration curves (10-bin stratification). Decision curve analysis (DCA) was performed to evaluate the clinical net benefit across a range of threshold probabilities. Descriptive model comparisons based on AUC, Brier score, area under the precision-recall curve (AUPRC, estimated using average precision), and a composite score were summarized as heatmaps. For each model, the classification threshold was selected by maximizing the Youden index using the training cohort’s predicted probabilities and observed PNI status. The full-precision training-derived threshold was applied unchanged to the internal test cohort and both external validation cohorts, with no re-optimization in these cohorts. Feature importance was interpreted using SHAP (SHapley Additive exPlanations) values derived from the random forest model on the internal validation set. Both global feature importance (SHAP summary bar plot) and individual feature effect analyses (SHAP scatter plot) were generated. Statistical significance was defined as a two-sided *p* < 0.05.

## 3 Results

### 3.1 Patient characteristics

A total of 205 patients with pathologically confirmed pancreatic cancer were included, comprising 137 (66.8%) with PNI and 68 (33.2%) without PNI (Figure 2). Of these, 143 patients from Centers I and II were divided into training (*n* = 100; 66 PNI-positive) and internal test (*n* = 43; 28 PNI-positive) sets. The first and second external validation cohorts included 28 patients (19 with PNI and 9 without PNI) and 34 patients (24 with PNI and 10 without PNI), respectively. The mean age was 58.42 ± 10.16 years, and 117 patients (57.1%) were male. Compared with patients without PNI, those with PNI more frequently had tumors in the pancreatic head (65.0% vs. 42.6%, *p* = 0.004) and advanced cT-stage (T3/4: 45.3% vs. 29.4%, *p* = 0.042). Other baseline characteristics did not differ significantly between the groups (Table 1).

**Table 1:** Baseline characteristics of patients with pancreatic cancer according to perineural invasion status.

Characteristic	Total (*N* = 205)	Non-PNI (*n* = 68)	PNI (*n* = 137)	Statistic	*p* Value
Age, years	58.42 ± 10.16	58.21 ± 9.47	58.53 ± 10.52	*t* = −0.212	0.833
Gender, *n* (%)				*χ*^2^ = 0.984	0.321
Male	117 (57.1)	35 (51.5)	82 (59.9)		
Female	88 (42.9)	33 (48.5)	55 (40.1)		
Smoking, *n* (%)				*χ*^2^ = 0.000	0.989
No	131 (63.9)	44 (64.7)	87 (63.5)		
Yes	74 (36.1)	24 (35.3)	50 (36.5)		
Drinking, *n* (%)				*χ*^2^ = 0.007	0.932
No	150 (73.2)	49 (72.1)	101 (73.7)		
Yes	55 (26.8)	19 (27.9)	36 (26.3)		
Family history of tumor, *n* (%)				—	1.000
No	195 (95.1)	65 (95.6)	130 (94.9)		
Yes	10 (4.9)	3 (4.4)	7 (5.1)		
Chronic pancreatitis, *n* (%)				—	1.000
No	193 (94.1)	64 (94.1)	129 (94.2)		
Yes	12 (5.9)	4 (5.9)	8 (5.8)		
Diabetes, *n* (%)				*χ*^2^ = 0.000	0.983
No	143 (69.8)	48 (70.6)	95 (69.3)		
Yes	62 (30.2)	20 (29.4)	42 (30.7)		
CA19-9, U/mL	386.06 ± 652.24	360.40 ± 521.69	398.79 ± 709.61	*t* = −0.438	0.662
CEA, ug/L	5.70 ± 8.68	4.95 ± 4.28	6.08 ± 10.17	*t* = −1.113	0.267
CA125, U/mL	30.93 ± 61.18	37.75 ± 68.48	27.54 ± 57.18	*t* = 1.059	0.292
Maximum tumor diameter, cm	3.30 (2.50–4.50)	3.00 (2.00–4.50)	3.50 (2.70–4.50)	*U* = 3912.5	0.062
Tumor location, *n* (%)				*χ*^2^ = 8.374	**0.004**
Pancreatic head	118 (57.6)	29 (42.6)	89 (65.0)		
Pancreatic body or/and tail	87 (42.4)	39 (57.4)	48 (35.0)		
cT-stage, *n* (%)				*χ*^2^ = 4.116	**0.042**
T1 or T2	123 (60.0)	48 (70.6)	75 (54.7)		
T3 or T4	82 (40.0)	20 (29.4)	62 (45.3)		
N stage, *n* (%)				*χ*^2^ = 0.399	0.527
N0	131 (63.9)	46 (67.6)	85 (62.0)		
N1 or N2	74 (36.1)	22 (32.4)	52 (38.0)		

Age, CA19-9, CEA, and CA125 are presented as mean ± SD; maximum tumor diameter as median (IQR); and categorical variables as *n* (%). PNI, perineural invasion; IQR, interquartile range; SD, standard deviation. Two-sided *p* values were obtained using an independent-samples Student’s *t*-test for age, Welch’s independent-samples *t*-tests for CA19-9, CEA, and CA125, a Mann–Whitney U test for maximum tumor diameter, and chi-square or Fisher’s exact tests for categorical variables. Bold *p* values indicate statistically significant differences.

### 3.2 Independent predictors of PNI

Univariable logistic regression identified tumor location and cT-stage as factors associated with PNI. Tumors in the pancreatic body and/or tail had lower odds of PNI than those in the pancreatic head (OR = 0.40, 95% CI: 0.22–0.73, *p* = 0.003), whereas advanced cT-stage (T3/4 vs. T1/2) was associated with higher odds (OR = 1.98, 95% CI: 1.07–3.69, *p* = 0.03). Both variables remained independently associated with PNI in the multivariable model: the adjusted OR was 0.36 (95% CI: 0.20–0.67, *p* = 0.001) for tumor location and 2.26 (95% CI: 1.18–4.30, *p* = 0.01) for cT-stage (Table 2). These two variables were therefore retained as clinical predictors for model development.

**Table 2:** Univariable and multivariable logistic regression analysis of characteristics associated with perineural invasion.

Characteristic	Univariable analysis		Multivariable analysis		
	OR (95% CI)	*p* value	*β* Coefficient	OR (95% CI)	*p* value
Age, years	1.00 (0.97, 1.03)	0.83	—	—	—
Gender					
Male	Reference				
Female	0.71 (0.40, 1.28)	0.25	—	—	—
Smoking history					
No	Reference				
Yes	1.05 (0.57, 1.93)	0.87	—	—	—
Drinking history					
No	Reference				
Yes	0.92 (0.48, 1.76)	0.80	—	—	—
Family history of tumor					
No	Reference				
Yes	1.17 (0.29, 4.66)	0.83	—	—	—
Chronic pancreatitis					
No	Reference				
Yes	0.99 (0.29, 3.42)	0.99	—	—	—
Diabetes					
No	Reference				
Yes	1.06 (0.56, 2.00)	0.86	—	—	—
CA19-9	1.00 (1.00, 1.00)	0.69	—	—	—
CEA	1.02 (0.98, 1.06)	0.40	—	—	—
CA125	1.00 (0.99, 1.00)	0.29	—	—	—
Maximum tumor diameter	1.09 (0.91, 1.29)	0.35	—	—	—
Tumor location					
Pancreatic head	Reference				
Pancreatic body or/and tail	0.40 (0.22, 0.73)	**0.003**	−1.01	0.36 (0.20, 0.67)	**0.001**
cT-stage					
T1 or T2	Reference				
T3 or T4	1.98 (1.07, 3.69)	**0.03**	0.81	2.26 (1.18, 4.30)	**0.01**
N stage					
N0	Reference				
N1 or N2	1.28 (0.69, 2.36)	0.43	—	—	—

Data in parentheses are 95% CIs. CI, confidence interval; PNI, perineural invasion. Variables with *p* < 0.2 in univariable analysis were entered into multivariable analysis using forward stepwise selection (*p*-enter = 0.10, *p*-remove = 0.15). Bold *p* values indicate statistically significant differences.

### 3.3 Feature selection

After the two-stage feature selection process, a total of 9 ITH-related features were retained across the three MRI sequences. For the T2WI sequence, 5 features were selected: 3D_T2WI_Max_Subregion_Proportion, 2D_T2WI_ITHscore_K6, 3D_T2WI_MIR, 3D_T2WI_FDVarIndex, and 3D_T2WI_IHI. For the DWI sequence, 1 feature was retained: 2D_DWI_ITHscore_K3. For the T1CE sequence, 3 features were selected: 3D_T1CE_MIR, 3D_T1CE_Max_Min_Volume_Ratio, and 3D_T1CE_VolWeightedFD.

### 3.4 Performance of machine learning models

Single-sequence model performance. Eleven machine learning algorithms were trained and evaluated on each individual MRI sequence across four datasets. Across all single-sequence models, T2WI-based models generally demonstrated the highest diagnostic performance, followed by T1CE and DWI models (Supplementary Figure S1; Supplementary Tables S2–S4). For T2WI-based models, the highest internal test AUC was 0.723 for DT. In the first external cohort, LightGBM and LDA shared the highest displayed AUC of 0.789; in the second, AdaBoost achieved 0.788. The highest DWI AUCs in the internal, first external, and second external cohorts were 0.596 (KNN), 0.623 (LightGBM), and 0.790 (KNN), respectively; corresponding T1CE maxima were 0.714 (KNN), 0.749 (LDA), and 0.767 (AdaBoost). These are descriptive maxima across algorithms, rather than performance estimates for one prespecified model.

Multi-sequence combined model performance. Combined-sequence models integrating features from T2WI, DWI, and T1CE exhibited substantially improved performance compared to individual single-sequence models across all datasets (Figure 3; Supplementary Table S5). In the training set, combined models achieved AUC values ranging from 0.875 (NB) to 0.999 (KNN, GBDT, AdaBoost). RF had the highest internal test AUC of 0.881, followed by LDA (0.876) and LR (0.871). RF also had the highest AUCs in the first and second external validation cohorts: 0.860 and 0.850, respectively.

**Figure 3:**
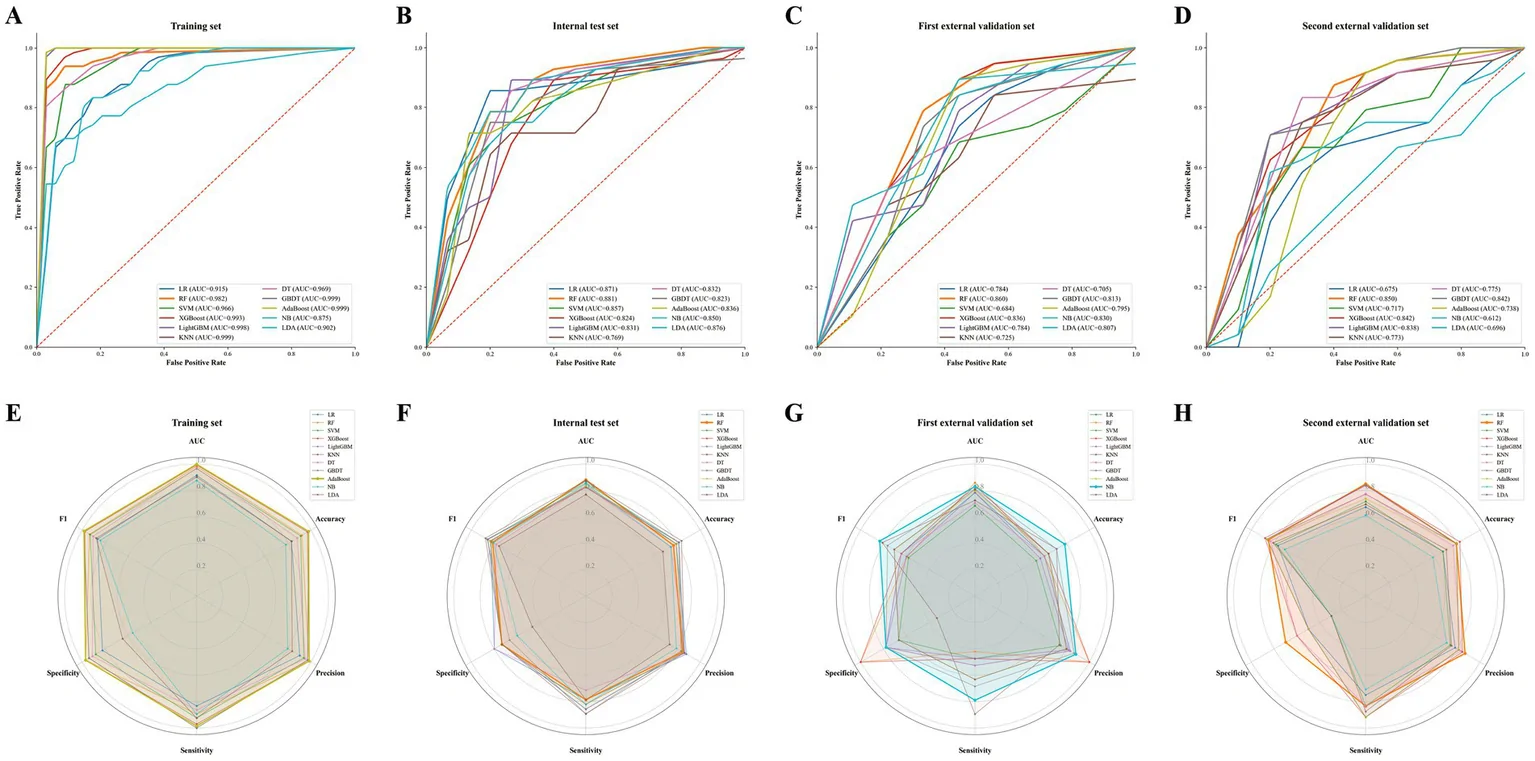
Performance of machine learning models integrating T2WI, DWI, and T1CE sequences in identifying perineural invasion status of pancreatic cancer. **(A,E)** Receiver operating characteristic (ROC) curves and radar charts in the training set. **(B,F)** ROC curves and radar charts in the internal test set. **(C,G)** ROC curves and radar charts in the first external validation set. **(D,H)** ROC curves and radar charts in the second external validation set.

These results indicate that multi-sequence integration consistently enhanced model robustness and generalizability compared with single-sequence approaches, with RF emerging as the algorithm with the most stable performance across all four datasets.

### 3.5 Comparison of clinical, ITH, and combined models

We further compared three categories of prediction models: clinical model (tumor location and cT-stage), ITH model (nine multi-sequence ITH features), and combined model (nine ITH features plus the two clinical predictors) (Figure 4; Supplementary Table S6).

**Figure 4:**
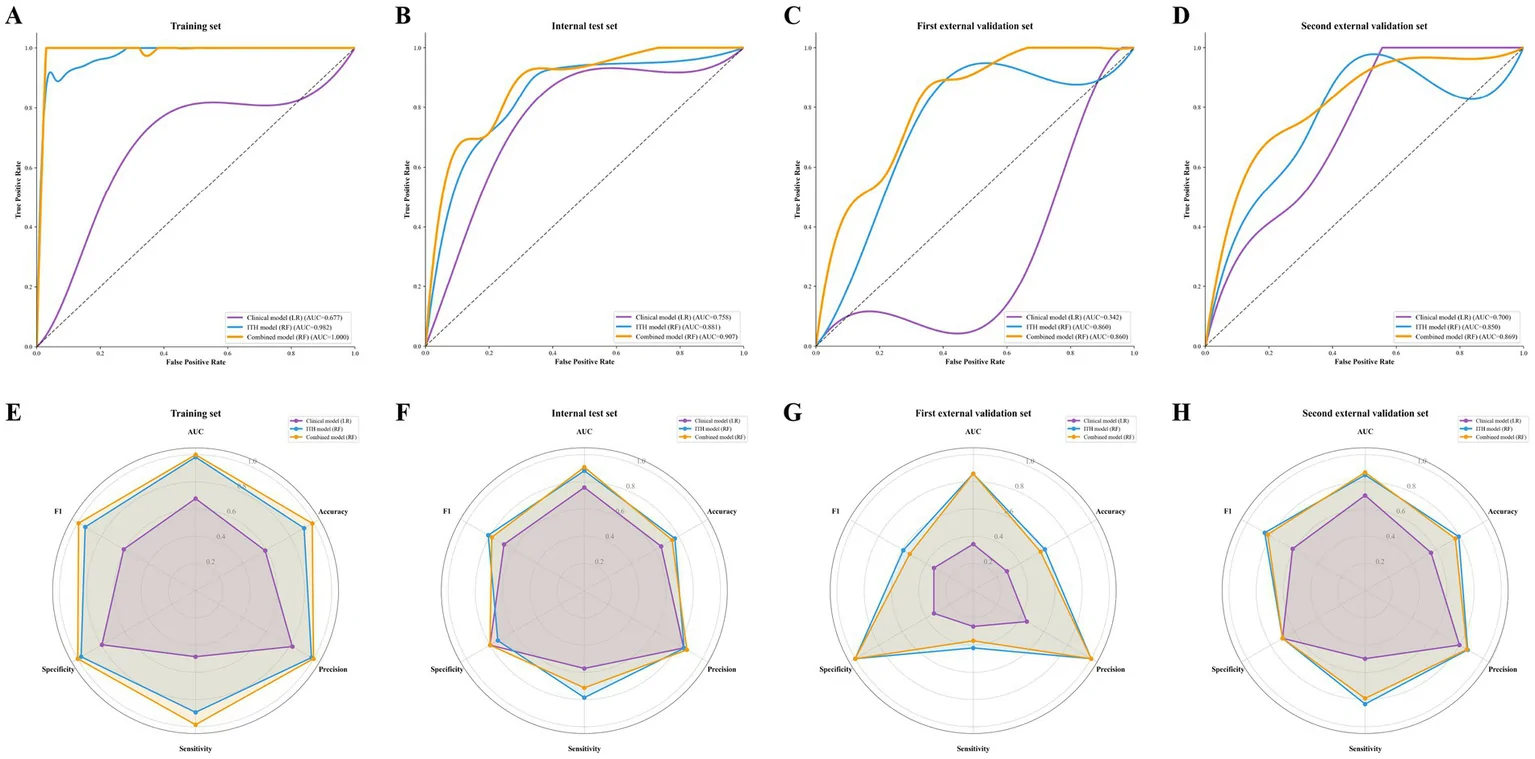
Performance of three different modality models in identifying perineural invasion status of pancreatic cancer. **(A,E)** Receiver operating characteristic (ROC) curves and radar charts in the training set. **(B,F)** ROC curves and radar charts in the internal test set. **(C,G)** ROC curves and radar charts in the first external validation set. **(D,H)** ROC curves and radar charts in the second external validation set.

The clinical model achieved an AUC of 0.677 in the training set and 0.758 in the internal test set, with external AUCs of 0.342 and 0.700, respectively, indicating particularly poor discrimination in the first external cohort. The ITH model demonstrated substantially higher AUC values: 0.982 (training), 0.881 (internal), 0.860 (first external), and 0.850 (second external). The combined model achieved the highest AUC in the training set (1.000) and maintained competitive performance in the internal test set (0.907), first external validation set (0.860), and second external validation set (0.869).

Decision curve analysis revealed that both the ITH and combined models provided substantially greater clinical net benefit than the clinical model across the majority of threshold probabilities in all four datasets (Figures 5A–D). Calibration varied across cohorts, with greater uncertainty in the small external validation cohorts (Figures 5E–H). Pairwise comparison heatmaps confirmed that the combined model consistently outperformed the clinical model across all evaluation metrics, while showing comparable or slightly improved performance relative to the ITH model (Supplementary Figure S2).

**Figure 5:**
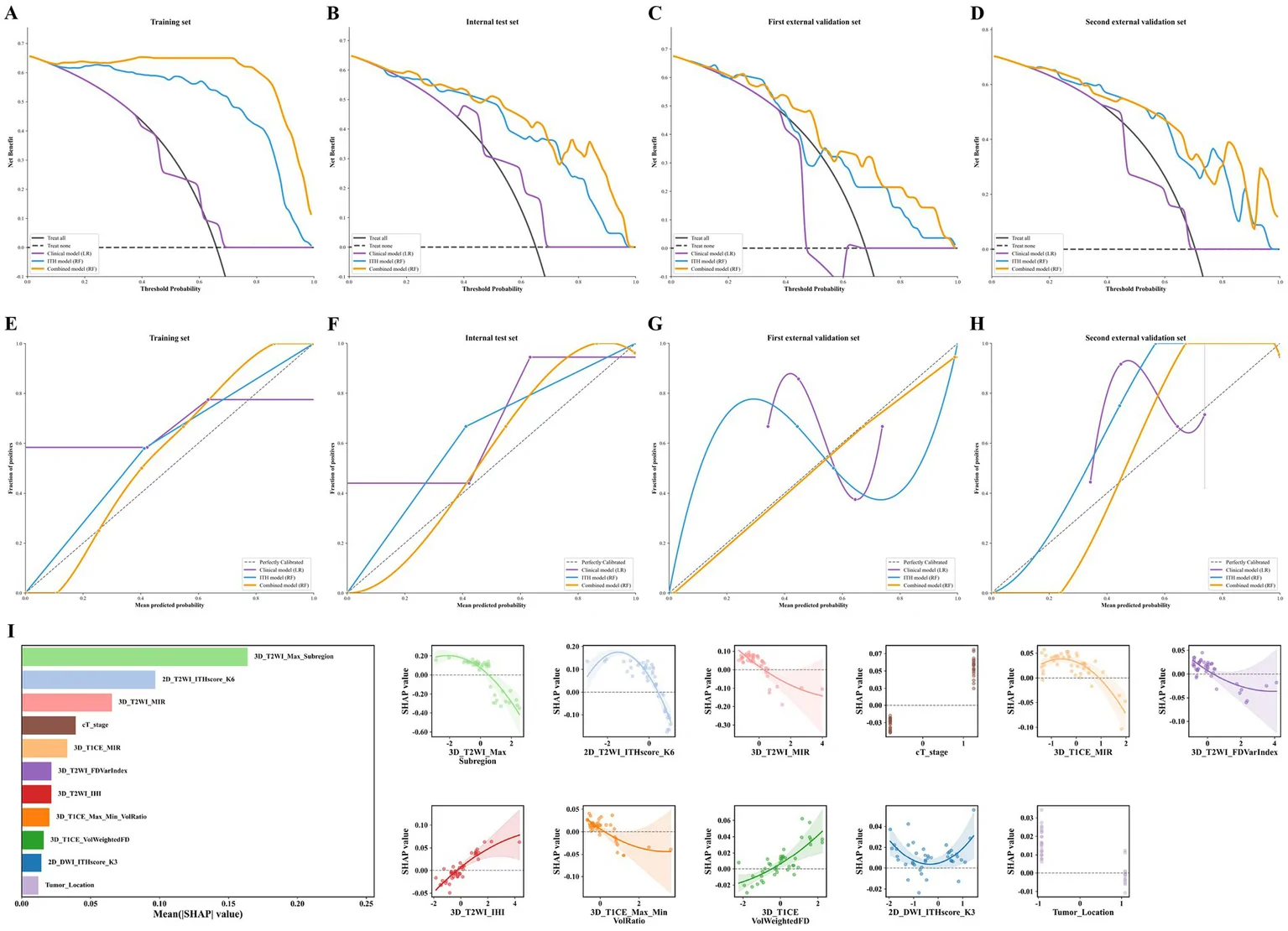
Evaluation and interpretability of three different modality models in identifying perineural invasion status of pancreatic cancer. Decision curves (DCs) of three different modality models in the training set **(A)**, internal test set **(B)**, first external validation set **(C)**, and second external validation set **(D)**. Calibration curves (CCs) in the training set **(E)**, internal test set **(F)**, first external validation set **(G)**, and second external validation set **(H)**. The bar plot in **(I)** ranks features of the combined RF model by mean absolute SHAP value; the accompanying dependence plots show standardized feature values against their SHAP contributions to the PNI model output.

### 3.6 Model interpretability

SHAP analysis was performed on the combined model to identify the most influential features driving PNI prediction. The three largest mean absolute SHAP values corresponded to 3D_T2WI_Max_Subregion, 2D_T2WI_ITHscore_K6, and 3D_T2WI_MIR, followed by cT-stage and 3D_T1CE_MIR; tumor location had the smallest contribution. SHAP summary bar plots revealed the global feature importance hierarchy, while SHAP scatter plots illustrated the relationship between individual feature values and their contribution to model output (Figure 5I). These interpretability analyses demonstrated that the combined model leveraged both imaging-derived heterogeneity features and clinicopathological characteristics in a complementary manner, providing biologically plausible and clinically interpretable predictions. Figure 6 illustrates the practical application of the joint model for preoperative discrimination of PNI status in pancreatic cancer. Representative PNI-positive and PNI-negative cases are presented, depicting the full pipeline from multi-sequence MRI acquisition through ITH subregion segmentation to final model output. The model generates a PNI probability score for each case, with higher ITHscores reflecting greater spatial fragmentation of tumor subregions and predicting PNI-positive status, consistent with the notion that pronounced intratumoral heterogeneity mirrors the complex tumor-stroma-neural interactions driving perineural spread. Conversely, tumors exhibiting more homogeneous imaging phenotypes with lower ITHscores are classified as PNI-negative.

**Figure 6:**
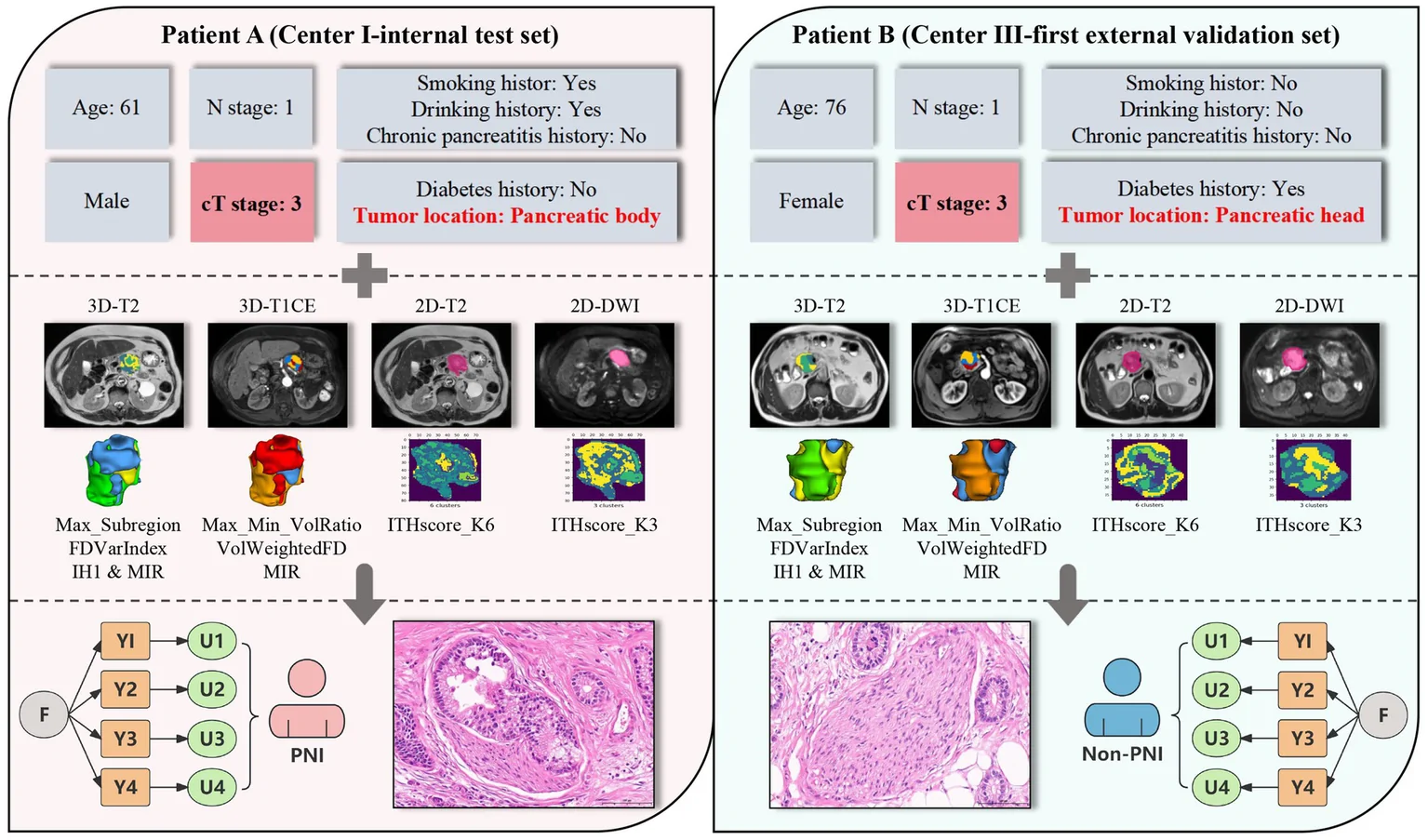
Practical application of the joint model in discriminating perineural invasion status of pancreatic cancer.

## 4 Discussion

In this multi-center study, we developed and externally validated a multi-sequence MRI-based machine learning framework for preoperative PNI prediction in PDAC. The combined clinical–ITH model achieved AUCs of 0.860 and 0.869 in the two independent external validation cohorts, compared with 0.860 and 0.850 for the ITH model and 0.342 and 0.700 for the clinical model. The internal test AUCs were 0.907, 0.881, and 0.758, respectively. Random forest demonstrated the most stable performance across all cohorts. To our knowledge, this is the first study to apply a spatial ITH scoring framework across multiple MRI sequences for PNI prediction in PDAC, validated in a four-center design.

Prior CT-based radiomics models for PNI prediction have reported single-center AUCs of 0.803–0.881 (Lan et al., 2026; Sun et al., 2025), while an interpretable fusion deep learning model on super-resolution MRI achieved AUCs of 0.929 (internal) and 0.832 (external) (Li et al., 2026a). Our ITH model achieved comparable external validation performance (0.850–0.860), while offering inherent interpretability: unlike opaque deep learning features, our spatial clustering–derived ITH features explicitly quantify the fragmentation and architectural complexity of intratumoral subregions, providing a transparent link between imaging heterogeneity and the clonal architecture hypothesized to underlie PNI. Additionally, validation across different MRI platforms supports the cross-center predictive performance of the framework, although direct assessment of scanner-related feature reproducibility remains necessary (Huang et al., 2023). The combination of explicit spatial descriptors and random forest provides a practical basis for further clinical translation (Kelly et al., 2019; Topol, 2019).

The biological plausibility of ITH-based PNI prediction rests on the well-established relationship between spatial heterogeneity and aggressive tumor behavior. Spatial ITH reflects the coexistence of distinct subclones driven by Darwinian evolutionary dynamics within the desmoplastic stroma (McGranahan and Swanton, 2017). In PDAC, the dense stroma creates spatially heterogeneous niches that exert differential selective pressures on subclones (Öhlund et al., 2017). Neurotrophic signaling and peripheral nerve remodeling are implicated in tumor–nerve interactions associated with PNI (Gu et al., 2025; Liu et al., 2026). The spatial fragmentation quantified by our ITHscore may thus serve as a noninvasive imaging surrogate for the clonal diversity and architectural complexity characterizing PNI-prone tumors, consistent with radiogenomic evidence that imaging phenotypes reflecting angiogenesis and inflammation are spatially anti-correlated within tumors (Su et al., 2023), and with the broader finding that high ITH is consistently associated with treatment resistance and poor prognosis across cancer types (Alizadeh et al., 2015; Chen et al., 2026).

Multi-sequence integration consistently outperformed any single sequence in our study. T2WI-based models demonstrated the highest single-sequence performance, likely owing to the sensitivity of T2WI to tissue compositional changes—edema, fibrosis, and inflammatory infiltration—that accompany perineural spread. DWI contributed unique information by probing microstructural integrity at the invasive front (Palombo et al., 2026), while T1CE captured perfusion heterogeneity associated with desmoplastic reaction (Hoffmann et al., 2024; Ramtohul et al., 2024). This multi-sequence advantage aligns with growing recognition that quantitative multi-parametric imaging provides more comprehensive tumor microenvironment characterization than any single modality (Hoffmann et al., 2024), and with emerging evidence that multi-parametric spatial profiling can reveal determinants of disease progression (Bojmar et al., 2024; Lin et al., 2023).

SHAP-based interpretability analysis identified nine ITH features as the primary contributors to model predictions. The 2D and 3D ITH scores, computed by partitioning the tumor into *K* = 3 or 6 subregions through K-means clustering, quantified the spatial fragmentation of tumor architecture. Among these, the 3D_Max_Subregion feature, which represents the proportion of the largest connected cluster component relative to total tumor volume, was a top contributor. In the SHAP dependence plot, higher values of this feature were generally associated with lower SHAP contributions to the model output for PNI (Figure 5I). Thus, a larger connected subregion was associated with a lower model contribution to PNI prediction. Although clonal selection provides a conceptual framework for understanding tumor heterogeneity (Black and McGranahan, 2021), this imaging association does not establish a specific clonal mechanism or neural tropism. The result should be interpreted as a model-specific imaging association requiring biological validation, alongside prior evidence for the predictive value of spatial ITH descriptors (Shi et al., 2023).

The T1CE-derived features (3D_T1CE_MIR and 3D_T1CE_VolWeightedFD) provided complementary predictive information. MIR captures the full dynamic range of signal intensity, reflecting coexistence of regions with markedly different enhancement patterns, which is a hallmark of vascular and stromal heterogeneity arising from aberrant neovascularization in PNI-positive tumors (Lin et al., 2026). The volume-weighted fractal dimension quantifies spatial complexity of subregion boundaries; higher values indicate more irregular boundaries, potentially reflecting disorganized infiltrative growth characteristic of perineural spread (Lopes and Betrouni, 2009).

Clinical variables provided a modest, cohort-dependent contribution to the ITH model. Adding tumor location and cT-stage increased the AUC from 0.881 to 0.907 in the internal test set and from 0.850 to 0.869 in the second external cohort, while the first external AUC remained 0.860. The clinical model yielded an AUC of 0.342 in the first external cohort, whereas both imaging-based models achieved 0.860 in the same patients. This distinction supports the predictive contribution of MRI-derived ITH features beyond the two clinical variables. The low clinical-model AUC may reflect sampling variability or differences in case mix; differences in cT-stage assessment or documentation are also possible, but cannot be established from this result alone.

Several limitations should be acknowledged. First, the retrospective cohort included only surgically resected patients, providing a histopathological reference standard for PNI but limiting extrapolation to patients who did not undergo resection. Borderline resectable disease was not analyzed separately, and validation in this clinically important population is needed. Second, manual VOI delineation requires radiologist time and limits scalability. Inter-observer feature screening (ICC > 0.80) supports reproducibility, but automated segmentation was not evaluated; nnU-Net is a potential direction for workflow development (Isensee et al., 2021). Third, scanner manufacturers and acquisition protocols varied across centers. Resampling, N4 correction, and Z-score standardization addressed selected sources of variation, but no ComBat or other feature-level batch-effect correction was applied. Although the ITH and combined models maintained external AUCs of 0.850–0.869, residual scanner effects cannot be excluded, and the value of additional harmonization warrants evaluation (Huchthausen et al., 2026). Fourth, the overall sample size was modest, particularly in the two external cohorts (*n* = 28 and *n* = 34), which limits the precision of performance estimates. Calibration and the performance of the fixed thresholds require further validation in larger independent cohorts. Finally, the biological mechanisms linking specific ITH patterns to PNI remain incompletely understood; integrated multi-omic studies correlating imaging heterogeneity with spatial profiling techniques would provide deeper mechanistic insight (Zhou et al., 2026).

## 5 Conclusion

We developed a multi-sequence MRI-based framework integrating 2D and 3D intratumoral heterogeneity features with machine learning for preoperative PNI prediction in PDAC. The combined clinical–ITH model achieved AUCs of 0.860 and 0.869 in two independent external cohorts, supporting its predictive value across participating centers. Spatial ITH descriptors and SHAP analysis provide interpretable information on the imaging features contributing to PNI prediction. These findings support the potential of MRI-based ITH assessment for preoperative risk stratification. Further validation in broader clinical populations, together with evaluation of automated segmentation and scanner harmonization, will facilitate clinical translation.

## Data Availability

The raw data supporting the conclusions of this article will be made available by the authors, without undue reservation.
