# Supplementary material for: Machine learning models integrating 2D and 3D intratumoral heterogeneity features from multi-sequence MRI: accurate prediction of perineural invasion in pancreatic ductal adenocarcinoma
Source: Front Artif Intell. 2026 Oct 20;9:1975752. doi: 10.3389/frai.2026.1975752 (PMC13727598; doi:10.3389/frai.2026.1975752)
Supplement: Supplementary file 1 [file Data_Sheet_1.zip › Supplementary Tables/Table 1.DOCX]

| **Table S1 The protocol of MRI scanning in four centers** | | | | | | | | | | | | | |
| --- | --- | --- | --- | --- | --- | --- | --- | --- | --- | --- | --- | --- | --- |
| **Center** | **Machine model** | **Magnetic field (T)** | **Coil** | **FOV**  **(mm×mm)** | **T2WI** | | | **DWI** | | | **T1CE** | | |
|  |  |  |  |  | **Thickness (mm)** | **Spacing**  **(mm)** | **TR/TE**  **(ms/ms)** | **Thickness (mm)** | **Spacing (mm)** | **b-value (s/mm²)** | **Thickness (mm)** | **Spacing**  **(mm)** | **TR/TE**  **(ms/ms)** |
|  |  |  |  |  |  |  |  |  |  |  |  |  |  |
| I | Philips·Ingenia | 1.5 | Abdominal phased array coil | 400×400 | 6.0 | 0.6 | 2000/90 | 6.0 | 1.0 | 50/800 | 3.0 | 0.5 | 4.0/1.96 |
|  | Philips·Elition | 3.0 | Abdominal phased array coil | 360×360 | 6.0 | 0.6 | 2000/80 | 6.0 | 0.5 | 50/800 | 2.0 | 1.0 | 3.6/1.32 |
|  | Siemens·Altea | 1.5 | Abdominal phased array coil | 380×380 | 6.0 | 0.6 | 2490/98 | 5.0 | 1.0 | 50/800 | 2.0 | 1.0 | 164/2.38 |
| II | Philips·Achieva | 3.0 | Abdominal phased array coil | 400×400 | 6.0 | 1.0 | 1300/100 | 6.0 | 1.0 | 50/800 | 2.0 | 1.0 | 3.1/1.51 |
|  | GE·Discovery 750W | 3.0 | Adaptive image receive coil | 380×380 | 6.0 | 1.0 | 1400/80 | 5.0 | 1.0 | 50/800 | 2.0 | 1.0 | 4.2/2.0 |
| III | GE·Signa HDxt | 3.0 | Abdominal phased array coil | 512×512 | 7.0 | 0.7 | 9500/89 | 6.0 | 1.0 | 50/1000 | 2.0 | 0.5 | 3.85/1.67 |
|  | Siemens·MAGNETOM Prisma | 3.0 | Abdominal phased array coil | 420×420 | 6.0 | 0.6 | 9800/77 | 5.0 | 1.0 | 50/1000 | 2.0 | 0.5 | 3.97/1.29 |
|  | Siemens·MAGNETOM Aera | 1.5 | Abdominal phased array coil | 512×512 | 6.0 | 0.6 | 2900/84 | 5.0 | 1.0 | 50/1000 | 2.0 | 0.5 | 4.10/1.86 |
| IV | Siemens·Verio Dot | 1.5 | Total imaging matrix coil | 420×420 | 6.0 | 1.2 | 1200/92 | 5.0 | 1.0 | 50/800 | 3.0 | 0.6 | 4.74/2.28 |
|  | Siemens·Verio Dot | 3.0 | Total imaging matrix coil | 400×400 | 6.0 | 1.2 | 1400/109 | 5.0 | 0.5 | 50/800 | 3.0 | 0.6 | 400/75 |
|  | GE·Signa Premier XT | 3.0 | Adaptive image receive coil | 400×400 | 4.0 | 0.4 | 6666.7/86 | 6.5 | 1.0 | 50/800 | 2.2 | 1.1 | 3.4/1.6 |
| Note: FOV, field of vision; T, tesla; T2WI, T2 weighted imaging; DWI, diffusion-weighted imaging; T1CE, T1 weighted contrast enhancement; TE, echo time; TR, repetition time. | | | | | | | | | | | | | |
