# Supplementary material for: Machine learning models integrating 2D and 3D intratumoral heterogeneity features from multi-sequence MRI: accurate prediction of perineural invasion in pancreatic ductal adenocarcinoma
Source: Front Artif Intell. 2026 Oct 20;9:1975752. doi: 10.3389/frai.2026.1975752 (PMC13727598; doi:10.3389/frai.2026.1975752)
Supplement: Supplementary file 1 [file Data_Sheet_1.zip › Supplementary Tables/Table 2.DOCX]

| **Table S2 Performance of T2WI-based machine learning models in identifying perineural invasion status of pancreatic cancer** | | | | | | | |
| --- | --- | --- | --- | --- | --- | --- | --- |
| **Dataset** | **Model** | **AUC (95% CI)** | **ACC** | **Sensitivity** | **Specificity** | **Precision** | **F1** |
| Training set | LR | 0.964 (0.927-0.990) | 0.900 | 0.894 | 0.912 | 0.952 | 0.922 |
| Training set | RF | 0.988 (0.969-0.999) | 0.920 | 0.909 | 0.941 | 0.968 | 0.938 |
| Training set | SVM | 0.986 (0.966-0.998) | 0.930 | 0.955 | 0.882 | 0.940 | 0.947 |
| Training set | XGBoost | 0.998 (0.991-1.000) | 0.960 | 0.939 | 1.000 | 1.000 | 0.969 |
| Training set | LightGBM | 1.000 (1.000-1.000) | 0.990 | 0.985 | 1.000 | 1.000 | 0.992 |
| Training set | KNN | 1.000 (1.000-1.000) | 1.000 | 1.000 | 1.000 | 1.000 | 1.000 |
| Training set | DT | 0.972 (0.943-0.992) | 0.880 | 0.818 | 1.000 | 1.000 | 0.900 |
| Training set | GBDT | 1.000 (1.000-1.000) | 1.000 | 1.000 | 1.000 | 1.000 | 1.000 |
| Training set | AdaBoost | 1.000 (1.000-1.000) | 1.000 | 1.000 | 1.000 | 1.000 | 1.000 |
| Training set | NB | 0.926 (0.871-0.972) | 0.870 | 0.924 | 0.765 | 0.884 | 0.904 |
| Training set | LDA | 0.952 (0.901-0.986) | 0.880 | 0.939 | 0.765 | 0.886 | 0.912 |
| Internal test set | LR | 0.644 (0.486-0.817) | 0.605 | 0.679 | 0.467 | 0.704 | 0.691 |
| Internal test set | RF | 0.720 (0.544-0.871) | 0.721 | 0.786 | 0.600 | 0.786 | 0.786 |
| Internal test set | SVM | 0.696 (0.530-0.853) | 0.628 | 0.786 | 0.333 | 0.688 | 0.733 |
| Internal test set | XGBoost | 0.720 (0.542-0.884) | 0.674 | 0.750 | 0.533 | 0.750 | 0.750 |
| Internal test set | LightGBM | 0.685 (0.502-0.850) | 0.651 | 0.750 | 0.467 | 0.724 | 0.737 |
| Internal test set | KNN | 0.629 (0.443-0.804) | 0.605 | 0.821 | 0.200 | 0.657 | 0.730 |
| Internal test set | DT | 0.723 (0.562-0.880) | 0.698 | 0.750 | 0.600 | 0.778 | 0.764 |
| Internal test set | GBDT | 0.713 (0.523-0.884) | 0.698 | 0.821 | 0.467 | 0.742 | 0.780 |
| Internal test set | AdaBoost | 0.662 (0.485-0.831) | 0.651 | 0.821 | 0.333 | 0.697 | 0.754 |
| Internal test set | NB | 0.615 (0.438-0.783) | 0.628 | 0.821 | 0.267 | 0.676 | 0.742 |
| Internal test set | LDA | 0.682 (0.527-0.848) | 0.698 | 0.893 | 0.333 | 0.714 | 0.794 |
| First external validation set | LR | 0.760 (0.524-0.938) | 0.607 | 0.526 | 0.778 | 0.833 | 0.645 |
| First external validation set | RF | 0.766 (0.538-0.931) | 0.643 | 0.474 | 1.000 | 1.000 | 0.643 |
| First external validation set | SVM | 0.766 (0.539-0.932) | 0.607 | 0.632 | 0.556 | 0.750 | 0.686 |
| First external validation set | XGBoost | 0.749 (0.524-0.918) | 0.536 | 0.474 | 0.667 | 0.750 | 0.581 |
| First external validation set | LightGBM | 0.789 (0.589-0.947) | 0.607 | 0.526 | 0.778 | 0.833 | 0.645 |
| First external validation set | KNN | 0.760 (0.567-0.912) | 0.643 | 0.684 | 0.556 | 0.765 | 0.722 |
| First external validation set | DT | 0.746 (0.561-0.908) | 0.571 | 0.368 | 1.000 | 1.000 | 0.538 |
| First external validation set | GBDT | 0.751 (0.517-0.925) | 0.607 | 0.632 | 0.556 | 0.750 | 0.686 |
| First external validation set | AdaBoost | 0.722 (0.472-0.921) | 0.571 | 0.579 | 0.556 | 0.733 | 0.647 |
| First external validation set | NB | 0.743 (0.487-0.936) | 0.643 | 0.684 | 0.556 | 0.765 | 0.722 |
| First external validation set | LDA | 0.789 (0.561-0.957) | 0.643 | 0.632 | 0.667 | 0.800 | 0.706 |
| Second external validation set | LR | 0.704 (0.490-0.885) | 0.676 | 0.792 | 0.400 | 0.760 | 0.776 |
| Second external validation set | RF | 0.746 (0.542-0.933) | 0.765 | 0.875 | 0.500 | 0.808 | 0.840 |
| Second external validation set | SVM | 0.771 (0.591-0.929) | 0.676 | 0.833 | 0.300 | 0.741 | 0.784 |
| Second external validation set | XGBoost | 0.763 (0.577-0.924) | 0.735 | 0.833 | 0.500 | 0.800 | 0.816 |
| Second external validation set | LightGBM | 0.742 (0.548-0.906) | 0.735 | 0.833 | 0.500 | 0.800 | 0.816 |
| Second external validation set | KNN | 0.679 (0.481-0.867) | 0.706 | 0.875 | 0.300 | 0.750 | 0.808 |
| Second external validation set | DT | 0.779 (0.589-0.947) | 0.735 | 0.750 | 0.700 | 0.857 | 0.800 |
| Second external validation set | GBDT | 0.704 (0.491-0.889) | 0.676 | 0.833 | 0.300 | 0.741 | 0.784 |
| Second external validation set | AdaBoost | 0.788 (0.621-0.925) | 0.676 | 0.875 | 0.200 | 0.724 | 0.792 |
| Second external validation set | NB | 0.688 (0.458-0.889) | 0.647 | 0.792 | 0.300 | 0.731 | 0.760 |
| Second external validation set | LDA | 0.758 (0.545-0.929) | 0.676 | 0.875 | 0.200 | 0.724 | 0.792 |
| Note. AUC, area under the receiver operating characteristic curve, presented with 95% confidence interval (95% CI) calculated via bootstrapping (1,000 iterations); ACC, accuracy; LR, logistic regression; RF, random forest; SVM, support vector machine; XGBoost, extreme gradient boosting; LightGBM, light gradient boosting machine; KNN, k-nearest neighbors; DT, decision tree; GBDT, gradient boosting decision tree; AdaBoost, adaptive boosting; NB, naive Bayes; LDA, linear discriminant analysis. F1-score is the harmonic means of precision and sensitivity. | | | | | | | |
