# Supplementary material for: Machine learning models integrating 2D and 3D intratumoral heterogeneity features from multi-sequence MRI: accurate prediction of perineural invasion in pancreatic ductal adenocarcinoma
Source: Front Artif Intell. 2026 Oct 20;9:1975752. doi: 10.3389/frai.2026.1975752 (PMC13727598; doi:10.3389/frai.2026.1975752)
Supplement: Supplementary file 1 [file Data_Sheet_1.zip › Supplementary Tables/Table 3.DOCX]

| **Table S3 Performance of DWI-based machine learning models in identifying perineural invasion status of pancreatic cancer** | | | | | | | |
| --- | --- | --- | --- | --- | --- | --- | --- |
| **Dataset** | **Model** | **AUC (95% CI)** | **ACC** | **Sensitivity** | **Specificity** | **Precision** | **F1** |
| Training set | LR | 0.502 (0.382-0.625) | 0.570 | 0.606 | 0.500 | 0.702 | 0.650 |
| Training set | RF | 0.783 (0.697-0.869) | 0.740 | 0.818 | 0.588 | 0.794 | 0.806 |
| Training set | SVM | 0.391 (0.279-0.502) | 0.660 | 1.000 | 0.000 | 0.660 | 0.795 |
| Training set | XGBoost | 0.839 (0.761-0.916) | 0.760 | 0.788 | 0.706 | 0.839 | 0.812 |
| Training set | LightGBM | 0.772 (0.689-0.857) | 0.710 | 0.773 | 0.588 | 0.785 | 0.779 |
| Training set | KNN | 0.999 (0.995-1.000) | 0.980 | 1.000 | 0.941 | 0.971 | 0.985 |
| Training set | DT | 0.781 (0.696-0.861) | 0.680 | 0.636 | 0.765 | 0.840 | 0.724 |
| Training set | GBDT | 0.981 (0.948-1.000) | 0.890 | 1.000 | 0.676 | 0.857 | 0.923 |
| Training set | AdaBoost | 0.731 (0.649-0.822) | 0.660 | 1.000 | 0.000 | 0.660 | 0.795 |
| Training set | NB | 0.512 (0.394-0.640) | 0.660 | 1.000 | 0.000 | 0.660 | 0.795 |
| Training set | LDA | 0.502 (0.382-0.625) | 0.660 | 1.000 | 0.000 | 0.660 | 0.795 |
| Internal test set | LR | 0.507 (0.288-0.732) | 0.442 | 0.357 | 0.600 | 0.625 | 0.455 |
| Internal test set | RF | 0.400 (0.222-0.582) | 0.465 | 0.643 | 0.133 | 0.581 | 0.610 |
| Internal test set | SVM | 0.560 (0.369-0.738) | 0.651 | 1.000 | 0.000 | 0.651 | 0.789 |
| Internal test set | XGBoost | 0.486 (0.309-0.662) | 0.465 | 0.571 | 0.267 | 0.593 | 0.582 |
| Internal test set | LightGBM | 0.414 (0.250-0.604) | 0.395 | 0.464 | 0.267 | 0.542 | 0.500 |
| Internal test set | KNN | 0.596 (0.409-0.761) | 0.605 | 0.786 | 0.267 | 0.667 | 0.721 |
| Internal test set | DT | 0.511 (0.350-0.674) | 0.488 | 0.464 | 0.533 | 0.650 | 0.542 |
| Internal test set | GBDT | 0.436 (0.249-0.622) | 0.558 | 0.750 | 0.200 | 0.636 | 0.689 |
| Internal test set | AdaBoost | 0.421 (0.261-0.591) | 0.651 | 1.000 | 0.000 | 0.651 | 0.789 |
| Internal test set | NB | 0.269 (0.108-0.441) | 0.651 | 1.000 | 0.000 | 0.651 | 0.789 |
| Internal test set | LDA | 0.507 (0.288-0.732) | 0.651 | 1.000 | 0.000 | 0.651 | 0.789 |
| First external validation set | LR | 0.228 (0.007-0.476) | 0.357 | 0.421 | 0.222 | 0.533 | 0.471 |
| First external validation set | RF | 0.608 (0.351-0.852) | 0.607 | 0.684 | 0.444 | 0.722 | 0.703 |
| First external validation set | SVM | 0.573 (0.344-0.772) | 0.679 | 1.000 | 0.000 | 0.679 | 0.809 |
| First external validation set | XGBoost | 0.599 (0.365-0.841) | 0.571 | 0.684 | 0.333 | 0.684 | 0.684 |
| First external validation set | LightGBM | 0.623 (0.389-0.853) | 0.500 | 0.579 | 0.333 | 0.647 | 0.611 |
| First external validation set | KNN | 0.576 (0.333-0.796) | 0.607 | 0.684 | 0.444 | 0.722 | 0.703 |
| First external validation set | DT | 0.620 (0.375-0.852) | 0.536 | 0.579 | 0.444 | 0.688 | 0.629 |
| First external validation set | GBDT | 0.547 (0.294-0.817) | 0.643 | 0.789 | 0.333 | 0.714 | 0.750 |
| First external validation set | AdaBoost | 0.512 (0.231-0.803) | 0.679 | 1.000 | 0.000 | 0.679 | 0.809 |
| First external validation set | NB | 0.181 (0.000-0.428) | 0.679 | 1.000 | 0.000 | 0.679 | 0.809 |
| First external validation set | LDA | 0.228 (0.007-0.476) | 0.679 | 1.000 | 0.000 | 0.679 | 0.809 |
| Second external validation set | LR | 0.554 (0.321-0.800) | 0.618 | 0.625 | 0.600 | 0.789 | 0.698 |
| Second external validation set | RF | 0.642 (0.448-0.834) | 0.588 | 0.625 | 0.500 | 0.750 | 0.682 |
| Second external validation set | SVM | 0.296 (0.096-0.514) | 0.706 | 1.000 | 0.000 | 0.706 | 0.828 |
| Second external validation set | XGBoost | 0.698 (0.509-0.866) | 0.706 | 0.708 | 0.700 | 0.850 | 0.773 |
| Second external validation set | LightGBM | 0.656 (0.458-0.830) | 0.647 | 0.708 | 0.500 | 0.773 | 0.739 |
| Second external validation set | KNN | 0.790 (0.600-0.949) | 0.794 | 0.917 | 0.500 | 0.815 | 0.863 |
| Second external validation set | DT | 0.604 (0.392-0.802) | 0.588 | 0.542 | 0.700 | 0.812 | 0.650 |
| Second external validation set | GBDT | 0.715 (0.466-0.919) | 0.735 | 0.875 | 0.400 | 0.778 | 0.824 |
| Second external validation set | AdaBoost | 0.548 (0.360-0.745) | 0.706 | 1.000 | 0.000 | 0.706 | 0.828 |
| Second external validation set | NB | 0.504 (0.296-0.727) | 0.706 | 1.000 | 0.000 | 0.706 | 0.828 |
| Second external validation set | LDA | 0.554 (0.321-0.800) | 0.706 | 1.000 | 0.000 | 0.706 | 0.828 |
| Note. AUC, area under the receiver operating characteristic curve, presented with 95% confidence interval (95% CI) calculated via bootstrapping (1,000 iterations); ACC, accuracy; LR, logistic regression; RF, random forest; SVM, support vector machine; XGBoost, extreme gradient boosting; LightGBM, light gradient boosting machine; KNN, k-nearest neighbors; DT, decision tree; GBDT, gradient boosting decision tree; AdaBoost, adaptive boosting; NB, naive Bayes; LDA, linear discriminant analysis. F1-score is the harmonic means of precision and sensitivity. | | | | | | | |
