# Supplementary material for: Machine learning models integrating 2D and 3D intratumoral heterogeneity features from multi-sequence MRI: accurate prediction of perineural invasion in pancreatic ductal adenocarcinoma
Source: Front Artif Intell. 2026 Oct 20;9:1975752. doi: 10.3389/frai.2026.1975752 (PMC13727598; doi:10.3389/frai.2026.1975752)
Supplement: Supplementary file 1 [file Data_Sheet_1.zip › Supplementary Tables/Table 4.DOCX]

| **Table S4 Performance of T1CE-based machine learning models in identifying perineural invasion status of pancreatic cancer** | | | | | | | |
| --- | --- | --- | --- | --- | --- | --- | --- |
| **Dataset** | **Model** | **AUC (95% CI)** | **ACC** | **Sensitivity** | **Specificity** | **Precision** | **F1** |
| Training set | LR | 0.670 (0.553-0.778) | 0.600 | 0.667 | 0.471 | 0.710 | 0.688 |
| Training set | RF | 0.932 (0.878-0.973) | 0.850 | 0.924 | 0.706 | 0.859 | 0.891 |
| Training set | SVM | 0.738 (0.614-0.852) | 0.670 | 1.000 | 0.029 | 0.667 | 0.800 |
| Training set | XGBoost | 0.966 (0.932-0.990) | 0.880 | 0.879 | 0.882 | 0.935 | 0.906 |
| Training set | LightGBM | 0.916 (0.859-0.962) | 0.840 | 0.818 | 0.882 | 0.931 | 0.871 |
| Training set | KNN | 0.999 (0.995-1.000) | 0.980 | 1.000 | 0.941 | 0.971 | 0.985 |
| Training set | DT | 0.788 (0.703-0.858) | 0.790 | 0.924 | 0.529 | 0.792 | 0.853 |
| Training set | GBDT | 0.996 (0.988-1.000) | 0.940 | 1.000 | 0.824 | 0.917 | 0.957 |
| Training set | AdaBoost | 0.920 (0.856-0.970) | 0.820 | 0.955 | 0.559 | 0.808 | 0.875 |
| Training set | NB | 0.675 (0.555-0.785) | 0.720 | 0.955 | 0.265 | 0.716 | 0.818 |
| Training set | LDA | 0.647 (0.524-0.761) | 0.720 | 0.955 | 0.265 | 0.716 | 0.818 |
| Internal test set | LR | 0.662 (0.464-0.824) | 0.628 | 0.714 | 0.467 | 0.714 | 0.714 |
| Internal test set | RF | 0.683 (0.474-0.869) | 0.744 | 0.821 | 0.600 | 0.793 | 0.807 |
| Internal test set | SVM | 0.493 (0.311-0.678) | 0.651 | 1.000 | 0.000 | 0.651 | 0.789 |
| Internal test set | XGBoost | 0.696 (0.509-0.862) | 0.628 | 0.643 | 0.600 | 0.750 | 0.692 |
| Internal test set | LightGBM | 0.637 (0.423-0.813) | 0.605 | 0.607 | 0.600 | 0.739 | 0.667 |
| Internal test set | KNN | 0.714 (0.533-0.847) | 0.674 | 0.893 | 0.267 | 0.694 | 0.781 |
| Internal test set | DT | 0.652 (0.488-0.787) | 0.674 | 0.821 | 0.400 | 0.719 | 0.767 |
| Internal test set | GBDT | 0.576 (0.388-0.755) | 0.558 | 0.679 | 0.333 | 0.655 | 0.667 |
| Internal test set | AdaBoost | 0.642 (0.440-0.814) | 0.698 | 0.821 | 0.467 | 0.742 | 0.780 |
| Internal test set | NB | 0.676 (0.502-0.826) | 0.674 | 0.929 | 0.200 | 0.684 | 0.788 |
| Internal test set | LDA | 0.660 (0.471-0.823) | 0.674 | 0.929 | 0.200 | 0.684 | 0.788 |
| First external validation set | LR | 0.684 (0.475-0.869) | 0.643 | 0.789 | 0.333 | 0.714 | 0.750 |
| First external validation set | RF | 0.450 (0.212-0.687) | 0.643 | 0.895 | 0.111 | 0.680 | 0.773 |
| First external validation set | SVM | 0.450 (0.220-0.688) | 0.679 | 1.000 | 0.000 | 0.679 | 0.809 |
| First external validation set | XGBoost | 0.450 (0.217-0.688) | 0.500 | 0.632 | 0.222 | 0.632 | 0.632 |
| First external validation set | LightGBM | 0.620 (0.379-0.837) | 0.536 | 0.526 | 0.556 | 0.714 | 0.606 |
| First external validation set | KNN | 0.444 (0.231-0.651) | 0.607 | 0.895 | 0.000 | 0.654 | 0.756 |
| First external validation set | DT | 0.415 (0.212-0.633) | 0.607 | 0.842 | 0.111 | 0.667 | 0.744 |
| First external validation set | GBDT | 0.450 (0.211-0.690) | 0.607 | 0.842 | 0.111 | 0.667 | 0.744 |
| First external validation set | AdaBoost | 0.401 (0.197-0.625) | 0.571 | 0.789 | 0.111 | 0.652 | 0.714 |
| First external validation set | NB | 0.632 (0.394-0.838) | 0.679 | 0.947 | 0.111 | 0.692 | 0.800 |
| First external validation set | LDA | 0.749 (0.550-0.928) | 0.679 | 0.947 | 0.111 | 0.692 | 0.800 |
| Second external validation set | LR | 0.383 (0.170-0.600) | 0.412 | 0.417 | 0.400 | 0.625 | 0.500 |
| Second external validation set | RF | 0.596 (0.356-0.852) | 0.735 | 0.833 | 0.500 | 0.800 | 0.816 |
| Second external validation set | SVM | 0.258 (0.057-0.479) | 0.676 | 0.958 | 0.000 | 0.697 | 0.807 |
| Second external validation set | XGBoost | 0.667 (0.437-0.899) | 0.735 | 0.792 | 0.600 | 0.826 | 0.809 |
| Second external validation set | LightGBM | 0.535 (0.287-0.798) | 0.676 | 0.750 | 0.500 | 0.783 | 0.766 |
| Second external validation set | KNN | 0.729 (0.559-0.899) | 0.765 | 0.917 | 0.400 | 0.786 | 0.846 |
| Second external validation set | DT | 0.613 (0.398-0.836) | 0.735 | 0.833 | 0.500 | 0.800 | 0.816 |
| Second external validation set | GBDT | 0.629 (0.383-0.875) | 0.765 | 0.917 | 0.400 | 0.786 | 0.846 |
| Second external validation set | AdaBoost | 0.767 (0.575-0.924) | 0.676 | 0.833 | 0.300 | 0.741 | 0.784 |
| Second external validation set | NB | 0.479 (0.273-0.700) | 0.618 | 0.875 | 0.000 | 0.677 | 0.764 |
| Second external validation set | LDA | 0.433 (0.212-0.661) | 0.618 | 0.833 | 0.100 | 0.690 | 0.755 |
| Note. AUC, area under the receiver operating characteristic curve, presented with 95% confidence interval (95% CI) calculated via bootstrapping (1,000 iterations); ACC, accuracy; LR, logistic regression; RF, random forest; SVM, support vector machine; XGBoost, extreme gradient boosting; LightGBM, light gradient boosting machine; KNN, k-nearest neighbors; DT, decision tree; GBDT, gradient boosting decision tree; AdaBoost, adaptive boosting; NB, naive Bayes; LDA, linear discriminant analysis. F1-score is the harmonic means of precision and sensitivity. | | | | | | | |
