# Supplementary material for: Machine learning models integrating 2D and 3D intratumoral heterogeneity features from multi-sequence MRI: accurate prediction of perineural invasion in pancreatic ductal adenocarcinoma
Source: Front Artif Intell. 2026 Oct 20;9:1975752. doi: 10.3389/frai.2026.1975752 (PMC13727598; doi:10.3389/frai.2026.1975752)
Supplement: Supplementary file 1 [file Data_Sheet_1.zip › Supplementary Tables/Table 5.DOCX]

| **Table S5 Performance of machine learning models integrating T2WI, DWI, and T1CE sequences in identifying perineural invasion status of pancreatic cancer** | | | | | | | |
| --- | --- | --- | --- | --- | --- | --- | --- |
| **Dataset** | **Model** | **AUC (95% CI)** | **ACC** | **Sensitivity** | **Specificity** | **Precision** | **F1** |
| Training set | LR | 0.915 (0.849-0.966) | 0.830 | 0.833 | 0.824 | 0.902 | 0.866 |
| Training set | RF | 0.982 (0.962-0.996) | 0.920 | 0.894 | 0.971 | 0.983 | 0.937 |
| Training set | SVM | 0.966 (0.928-0.993) | 0.910 | 0.924 | 0.882 | 0.938 | 0.931 |
| Training set | XGBoost | 0.993 (0.982-1.000) | 0.970 | 0.970 | 0.971 | 0.985 | 0.977 |
| Training set | LightGBM | 0.998 (0.994-1.000) | 0.980 | 0.985 | 0.971 | 0.985 | 0.985 |
| Training set | KNN | 0.999 (0.995-1.000) | 0.980 | 1.000 | 0.941 | 0.971 | 0.985 |
| Training set | DT | 0.969 (0.943-0.991) | 0.880 | 0.864 | 0.912 | 0.950 | 0.905 |
| Training set | GBDT | 0.999 (0.995-1.000) | 0.980 | 1.000 | 0.941 | 0.971 | 0.985 |
| Training set | AdaBoost | 0.999 (0.995-1.000) | 0.980 | 0.985 | 0.971 | 0.985 | 0.985 |
| Training set | NB | 0.875 (0.794-0.938) | 0.780 | 0.894 | 0.559 | 0.797 | 0.843 |
| Training set | LDA | 0.902 (0.830-0.958) | 0.830 | 0.924 | 0.647 | 0.836 | 0.878 |
| Internal test set | LR | 0.871 (0.744-0.963) | 0.791 | 0.786 | 0.800 | 0.880 | 0.830 |
| Internal test set | RF | 0.881 (0.752-0.972) | 0.767 | 0.786 | 0.733 | 0.846 | 0.815 |
| Internal test set | SVM | 0.857 (0.735-0.955) | 0.814 | 0.857 | 0.733 | 0.857 | 0.857 |
| Internal test set | XGBoost | 0.824 (0.669-0.950) | 0.791 | 0.821 | 0.733 | 0.852 | 0.836 |
| Internal test set | LightGBM | 0.831 (0.687-0.951) | 0.814 | 0.857 | 0.733 | 0.857 | 0.857 |
| Internal test set | KNN | 0.769 (0.614-0.899) | 0.674 | 0.786 | 0.467 | 0.733 | 0.759 |
| Internal test set | DT | 0.832 (0.676-0.953) | 0.744 | 0.714 | 0.800 | 0.870 | 0.784 |
| Internal test set | GBDT | 0.823 (0.660-0.951) | 0.814 | 0.893 | 0.667 | 0.833 | 0.862 |
| Internal test set | AdaBoost | 0.836 (0.694-0.953) | 0.791 | 0.821 | 0.733 | 0.852 | 0.836 |
| Internal test set | NB | 0.850 (0.729-0.951) | 0.744 | 0.821 | 0.600 | 0.793 | 0.807 |
| Internal test set | LDA | 0.876 (0.753-0.964) | 0.837 | 0.893 | 0.733 | 0.862 | 0.877 |
| First external validation set | LR | 0.784 (0.574-0.938) | 0.607 | 0.526 | 0.778 | 0.833 | 0.645 |
| First external validation set | RF | 0.860 (0.689-0.985) | 0.607 | 0.421 | 1.000 | 1.000 | 0.593 |
| First external validation set | SVM | 0.684 (0.450-0.870) | 0.536 | 0.474 | 0.667 | 0.750 | 0.581 |
| First external validation set | XGBoost | 0.836 (0.631-0.972) | 0.643 | 0.474 | 1.000 | 1.000 | 0.643 |
| First external validation set | LightGBM | 0.784 (0.549-0.947) | 0.571 | 0.474 | 0.778 | 0.818 | 0.600 |
| First external validation set | KNN | 0.725 (0.522-0.904) | 0.714 | 0.895 | 0.333 | 0.739 | 0.810 |
| First external validation set | DT | 0.705 (0.512-0.857) | 0.607 | 0.526 | 0.778 | 0.833 | 0.645 |
| First external validation set | GBDT | 0.813 (0.576-0.979) | 0.714 | 0.684 | 0.778 | 0.867 | 0.765 |
| First external validation set | AdaBoost | 0.795 (0.558-0.970) | 0.643 | 0.632 | 0.667 | 0.800 | 0.706 |
| First external validation set | NB | 0.830 (0.646-0.985) | 0.786 | 0.789 | 0.778 | 0.882 | 0.833 |
| First external validation set | LDA | 0.807 (0.606-0.957) | 0.643 | 0.632 | 0.667 | 0.800 | 0.706 |
| Second external validation set | LR | 0.675 (0.447-0.862) | 0.676 | 0.750 | 0.500 | 0.783 | 0.766 |
| Second external validation set | RF | 0.850 (0.689-0.973) | 0.794 | 0.833 | 0.700 | 0.870 | 0.851 |
| Second external validation set | SVM | 0.717 (0.500-0.896) | 0.676 | 0.833 | 0.300 | 0.741 | 0.784 |
| Second external validation set | XGBoost | 0.842 (0.675-0.968) | 0.824 | 0.917 | 0.600 | 0.846 | 0.880 |
| Second external validation set | LightGBM | 0.838 (0.668-0.971) | 0.794 | 0.917 | 0.500 | 0.815 | 0.863 |
| Second external validation set | KNN | 0.773 (0.580-0.950) | 0.794 | 0.917 | 0.500 | 0.815 | 0.863 |
| Second external validation set | DT | 0.775 (0.602-0.938) | 0.765 | 0.833 | 0.600 | 0.833 | 0.833 |
| Second external validation set | GBDT | 0.842 (0.676-0.968) | 0.794 | 0.917 | 0.500 | 0.815 | 0.863 |
| Second external validation set | AdaBoost | 0.738 (0.483-0.947) | 0.794 | 0.917 | 0.500 | 0.815 | 0.863 |
| Second external validation set | NB | 0.612 (0.390-0.822) | 0.588 | 0.708 | 0.300 | 0.708 | 0.708 |
| Second external validation set | LDA | 0.696 (0.476-0.868) | 0.706 | 0.875 | 0.300 | 0.750 | 0.808 |
| Note. AUC, area under the receiver operating characteristic curve, presented with 95% confidence interval (95% CI) calculated via bootstrapping (1,000 iterations); ACC, accuracy; LR, logistic regression; RF, random forest; SVM, support vector machine; XGBoost, extreme gradient boosting; LightGBM, light gradient boosting machine; KNN, k-nearest neighbors; DT, decision tree; GBDT, gradient boosting decision tree; AdaBoost, adaptive boosting; NB, naive Bayes; LDA, linear discriminant analysis. F1-score is the harmonic means of precision and sensitivity. | | | | | | | |
