# Supplementary material for: Machine learning models integrating 2D and 3D intratumoral heterogeneity features from multi-sequence MRI: accurate prediction of perineural invasion in pancreatic ductal adenocarcinoma
Source: Front Artif Intell. 2026 Oct 20;9:1975752. doi: 10.3389/frai.2026.1975752 (PMC13727598; doi:10.3389/frai.2026.1975752)
Supplement: Supplementary file 1 [file Data_Sheet_1.zip › Supplementary Tables/Table 6.DOCX]

| **Table S6 Comprehensive diagnostic metrics of three different modality models in identifying perineural invasion status of pancreatic cancer** | | | | | | | | | | | | |
| --- | --- | --- | --- | --- | --- | --- | --- | --- | --- | --- | --- | --- |
| **Dataset** | **Model** | **AUC** | **AUPRC** | **Sensitivity** | **Specificity** | **PPV** | **NPV** | **Youden Index** | **LR+** | **LR-** | **MCC** | **Best Threshold** |
| Training set | Clinical | 0.677 | 0.759 | 0.485 | 0.794 | 0.821 | 0.443 | 0.279 | 2.35 | 0.65 | 0.271 | 0.603 |
| Training set | ITH | 0.982 | 0.991 | 0.894 | 0.971 | 0.983 | 0.825 | 0.865 | 30.39 | 0.11 | 0.836 | 0.610 |
| Training set | Combined | 1.000 | 1.000 | 0.985 | 1.000 | 1.000 | 0.971 | 0.985 | - | 0.02 | 0.978 | 0.730 |
| Internal test set | Clinical | 0.758 | 0.802 | 0.571 | 0.800 | 0.842 | 0.500 | 0.371 | 2.86 | 0.54 | 0.356 | 0.603 |
| Internal test set | ITH | 0.881 | 0.936 | 0.786 | 0.733 | 0.846 | 0.647 | 0.519 | 2.95 | 0.29 | 0.506 | 0.610 |
| Internal test set | Combined | 0.907 | 0.951 | 0.714 | 0.800 | 0.870 | 0.600 | 0.514 | 3.57 | 0.36 | 0.491 | 0.730 |
| First external validation set | Clinical | 0.342 | 0.643 | 0.263 | 0.333 | 0.455 | 0.176 | -0.404 | 0.39 | 2.21 | -0.386 | 0.603 |
| First external validation set | ITH | 0.860 | 0.930 | 0.421 | 1.000 | 1.000 | 0.450 | 0.421 | - | 0.58 | 0.435 | 0.610 |
| First external validation set | Combined | 0.860 | 0.930 | 0.368 | 1.000 | 1.000 | 0.429 | 0.368 | - | 0.63 | 0.397 | 0.730 |
| Second external validation set | Clinical | 0.700 | 0.813 | 0.500 | 0.700 | 0.800 | 0.368 | 0.200 | 1.67 | 0.71 | 0.184 | 0.603 |
| Second external validation set | ITH | 0.850 | 0.929 | 0.833 | 0.700 | 0.870 | 0.636 | 0.533 | 2.78 | 0.24 | 0.519 | 0.610 |
| Second external validation set | Combined | 0.869 | 0.942 | 0.792 | 0.700 | 0.864 | 0.583 | 0.492 | 2.64 | 0.30 | 0.469 | 0.730 |
| Note. AUC, area under the receiver operating characteristic curve; AUPRC, area under the precision-recall curve; PPV, positive predictive value; NPV, negative predictive value; LR+, positive likelihood ratio; LR-, negative likelihood ratio; MCC, Matthews correlation coefficient; Best Threshold, optimal cutoff determined by the Youden index in training set. | | | | | | | | | | | | |
